# Erratum to: Quantifying Socioeconomic and Lifestyle Related Health Risks: Burden of Cardiovascular Disease Among Indian Males

**DOI:** 10.5195/cajgh.2015.237

**Published:** 2016-01-21

**Authors:** Neetu Purohit, Divya K. Bhati, Shiv D. Gupta, Azad S. Kundu

**Affiliations:** 1 Indian Institute of Health Management Research University;; 2 Indian Council of Medical Research

During the type-setting of the final version of the article,^1^ the title was misspelled on the website, page 2 of Word Document, and page 2 of PDF. The title was written as “Quantifying Socioeconomic and Lifestyle Related Health Risks: Burden of Cardiocascular Disease Among Indian Males” and the corrected title is “Quantifying Socioeconomic and Lifestyle Related Health Risks: Burden of Cardiovascular Disease Among Indian Males.”
